# Prevalence, Causes and Outcomes of Acute Gastrointestinal Bleeding in Rheumatoid Arthritis: A Systematic Review and Meta-Analysis

**DOI:** 10.31138/mjr.230324.pca

**Published:** 2024-06-30

**Authors:** Shobhit Piplani, Vladimir Jelic, Adejoke Johnson, Usman Shah, Shiny Kolli, Steve Kong, Nikola Tanasijevic, Vishal Reddy Bejugam, Sumaja Reddy Goguri, Phanidhar Mogga, Sripada Preetham Kasire, Salil Chaturvedi, Priyanshu Jain

**Affiliations:** 1 Jacobi Medical Centre/North Central Bronx, Albert Einstein College of Medicine, NYC Health and Hospitals, New York, New York, United States,; 2 Frank H. Netter MD School of Medicine/St. Vincent’s Medical Centre, Connecticut, United States,; 3 Jawaharlal Nehru Medical College, Belgaum, India

**Keywords:** gastrointestinal bleeding, rheumatoid arthritis, NSAIDs, autoimmune, prednisone

## Abstract

**Aim:**

The present study aims to investigate the prevalence, causes and outcomes of acute gastrointestinal (GI) bleeding in Rheumatoid arthritis (RA).

**Methods:**

A systemic search was conducted from electronic databases (PubMed/Medline, Cochrane Library, and Google Scholar) from inception to 14^th^ November 2023. All statistical analyses were conducted in Review Manager 5.4.1. Studies meeting inclusion criteria were selected. A random-effect model was used when heterogeneity was seen to pool the studies, and the result was reported in prevalence and their corresponding 95% confidence interval (CI). Other outcomes were assessed using qualitative analysis.

**Results:**

A total of eight studies (six observational studies and 2 trials were used to conduct this systematic review and meta-analysis. A total population of 138,041 patients was used. Pooled analysis showed a statistically significant risk of GI bleeding in RA patients receiving NSAIDs (prevalence = 2% (1%, 3%); P < 0.00001; I2 = 98%). Qualitatively, causes and outcomes were discussed.

**Conclusion:**

Our study showed that 2% RA patients were subjected to GI bleeding, when they used NSAIDs. Other causes of GI bleeding were age-related factors, cardiovascular events, history of GI complications, and peptic ulcers. Outcome varied by the use of specific NSAIDs and the presence of comorbidities. Recent guidelines for the management of RA may mention GI bleeding as a potential complication, but the level of emphasis placed on this issue varies. Some guidelines provide comprehensive recommendations for its prevention and management, while others offer limited guidance.

## INTRODUCTION

Rheumatoid arthritis is a debilitating autoimmune systemic inflammatory disorder that chronically affects joints and other soft tissues by causing widespread inflammation and structural damage.^1^ This rheumatological disorder is a significant cause of morbidity worldwide, affecting about 0.5% to 1% of the world population.^2^ It predominately causes destruction and damage to joints and cartilage which often leads to significant disability. Optimal management relies upon timely and accurate diagnosis which requires thorough clinical evaluation when encountering patients with risk factors and associated condition.^3^ With the passage of time the advancement in treatment models has evolved the awareness potential of gastrointestinal (GI) bleeding in patients with RA. From the 20th century with the introduction of disease-modifying antirheumatic drugs (DMARDs) to the early 2000s with the advent of biologic treatments and more recently the availability of Janus kinase (JAK) inhibitors, physicians have increasingly recognised the importance of monitoring and managing GI complications in RA. Notably, nonsteroidal anti-inflammatory drugs (NSAIDs), DMARDs, and corticosteroids have been associated with GI bleeding in RA patients.^4^

The diagnosis is based mainly on clinical features, supported by laboratory findings. Typically, the patients present with symmetrical polyarthritis involving mainly the small joints of hands and feet over weeks to months, associated with prolonged morning stiffness. This is often accompanied by systemic symptoms such as unexplained fever, fatigue, and loss of appetite.^5^ In addition to causing deterioration of the physical well-being of patients, rheumatoid arthritis also has a significant impact on the socioeconomic conditions of the affected individuals as well as the society as a whole.^6^ Failure to diagnose the condition and initiate effective management can lead to devastating complications that might involve major organ systems of the body including the respiratory, renal, gastrointestinal, nervous, and cardiovascular systems.^7^

GI involvement in RA can occur due to the disease itself, especially when long-term and poorly controlled, however, more often occurs due to iatrogenic causes secondary to NSAIDs, DMARDs, and biologics. Moreover, GI symptoms may occur as a result of associated conditions such as Sjogren and Felty syndromes. Symptoms include abdominal pain, heartburn, altered bowel habits, dyspepsia, dysphagia, nausea, vomiting, and GI bleeding.^8^ Gastrointestinal manifestations mainly occur due to rheumatoid vasculitis which is characterised by inflammation of small- and medium-sized blood vessels. This can lead to bowel necrosis, ischemic ulcers, gastritis, ischemic colitis, and other conditions associated with vessel inflammation which might manifest as severe and debilitating gastrointestinal symptoms.^9^

GI bleeding involves any bleeding that occurs anywhere from the oral cavity to the anus. Bleeding from the gastrointestinal tract can be categorised as upper GI bleed, small bowel bleed, and lower GI bleed. Although the aetiologies of gastrointestinal bleeding are diverse, the use of certain drugs, particularly NSAIDs, is a strong and consistent risk factor. Recent advancements in the use of endoscopic procedures for the diagnosis and management of GI bleeding have led to dramatic improvements in patients’ outcomes.^10^

Upper GI bleeding occurs due to various causes including peptic ulcer disease, esophagitis, gastritis, Mallory-Weiss tears, and cancer. Risk factors include previous episodes of upper GI bleed, use of certain drugs such as NSAIDs, chronic diseases, and old age. Upper GI bleed presents with melena or hematemesis often accompanied by other symptoms such as abdominal pain, syncope, dizziness, and light-headedness.^11^ Bleeding from the lower GI tract occurs mostly due to colonic pathologies such as diver-ticular disease, vascular malformations, polyps, colitis and malignancy. It is more common in the elderly, with diverticular haemorrhage being the most common aetiology.^12^ Different studies have shown that RA patients have an increased risk of serious upper as well as lower GI events including GI bleeding, and greater GI-related mortality as compared to normal individuals. However, the incidence of upper GI events such as bleeding, ulceration, obstruction, and perforation, but not the lower GI events, in RA patients has relatively declined over the decades. This decline is associated with the advancement of RA treatments, and the use of gastroprotective medicines such proton pump inhibitors. The development of disease-modifying antirheumatic medications (DMARDs) in the late twentieth century, followed by biologic therapy and, more recently, Janus kinase (JAK) inhibitors, has made RA management more effective in managing disease activity and lowering systemic inflammation.^13,14^ Some of the gastrointestinal manifestations are directly due to RA, but others occur either due to associated autoimmune disorders or as a result of therapy. These include peptic ulcer disease, gastritis, colitis, autoimmune hepatitis, and pancreatitis. The presentation is similar to that of other vasculitides that involve the GIT, especially polyarteritis nodosa (PAN). Ulceration may involve the oesophagus, stomach, small bowel, and large bowel, which may lead to bleeding.^15^

This systematic review and meta-analysis were performed to evaluate the occurrence of gastrointestinal bleeding in patients suffering from rheumatoid arthritis and to get a better understanding of the risk factors associated with it. This is to determine possible strategies for reducing the incidence of such incidents.

## METHODS

### Search strategy and databases

The systematic review was conducted following the guidelines of the Preferred Reporting Items for Systematic Reviews and Meta-analyses (PRISMA). An electronic search was conducted using PubMed/Medline, Cochrane Trial Register, and Google Scholar from their inception to 14^th^ November 2023. The following search string was used: (Acute Gastrointestinal haemorrhage OR bleeding) AND (rheumatoid arthritis OR RA). We additionally searched the referenced articles of previously published meta-analyses, cohort studies, and review articles to identify any relevant studies.

### Study selection criteria

Studies were selected if they followed our PECOS: P (Patients): Rheumatoid arthritis patients; E (Exposure): Gastrointestinal bleeding; C (Control): None; O (Outcomes): prevalence, causes and outcome of GI bleeding in RA patients; S (Studies): Observational studies and Randomised Controlled Trials.

### Data extraction and Quality assessment

The electronic databases were screened, and studies were exported to EndNote Reference Library version 20.0.1 (Clarivate Analytics, London, UK). The articles were then screened for the removal of duplicate articles The data extracted from the selected studies was exported to computer spreadsheet.

Quality assessment and bias assessment were done using New Ottawa Scale (NOS) score for observational studies and Cochrane Collaboration Tool for clinical trials. NOS score 1–5 was considered as high risk for bias, 6–7 was moderate, and score >7 was considered low risk of bias. Seven domains were assessed by The Cochrane Collaboration’s tool: adequate sequence generation, allocation concealment, blinding of participants and personnel, blinding of outcome assessment, incomplete outcome data, selective outcome reporting, and free of other bias. The individual domains and overall risk-of-bias judgment were expressed on one of three levels: high risk of bias, unclear risk of bias, and low risk of bias. Based on these factors, the overall quality of evidence was deemed as high, moderate, or low risk of bias.

### Statistical Analysis

Review Manager (version 5.4.1; Copenhagen: The Nordic Cochrane Centre, The Cochrane Collaboration, 2020) was used for all statistical analyses. The data from studies were pooled using a random-effects model when heterogeneity was seen. Analysis of results was done by calculating the inverse variance (IV) with respective 95% confidence intervals (CI). The chi-square test was performed to assess any differences between the subgroups. Sensitivity analysis was done to see if any individual study was driving the results and to implore reasons for high heterogeneity. As per the Cochrane Handbook, scale for heterogeneity was considered as follows: I2 = 25–60% – moderate; 50–90% – substantial; 75–100% – considerable heterogeneity, and P < 0.1 indicated significant heterogeneity.^2^ A P< 0.05 was considered significant for all analyses.

Prevalence was calculated through raw data. This along with other extracted information was used to find standard errors using the formula below, where “p” was the prevalence and “n” was the number of systemic lupus erythematosus (SLE) patients.

$$\text{SE}=\sqrt{\frac{\text{p}\times \left(1-\text{p}\right)}{n}}$$

The prevalence and standard error of each study were then input in the Review manager through the inverse variance method to compute pooled prevalence along with a 95% confidence interval.

Causes and outcomes of GI bleeding in RA patients were discussed qualitatively, using a narrative review approach.

## RESULTS

### Literature Search Results

**Figure 1.**
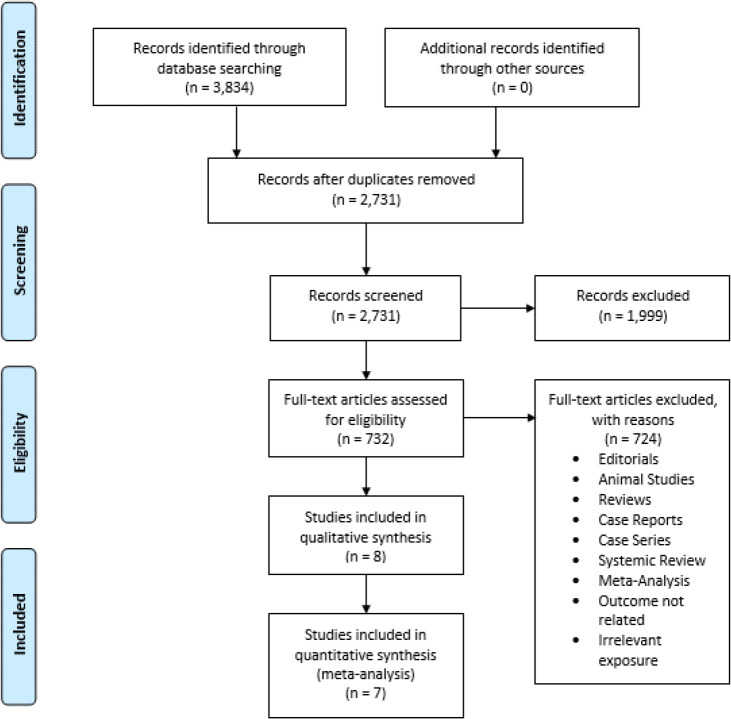
PRISMA flow diagram.

The initial literature search by using three electronic databases showed 3,834 research articles. After reading the title and abstract, 1,999 studies were excluded from the initial list. Seven studies were selected for quantitative analysis and eight for qualitative analysis after exclusion based on full text (n= 732). **Figure 1** shows the Prisma flow chart.

### Study Characteristics

**Table 1** provides the demographic and clinical data of the selected studies.^16–24^ The analysis included six cohort studies and two RCTs, with a patient population of 138,041. The average age of the patients in the included studies was 63.8 years. Although the sample sizes of some of the included studies are small these studies provide valuable insights regarding gastrointestinal complications in RA patients.

**Table 1.** Demographic and clinical characteristics of the included studies.

**Study**	**Year**	**Country**	**Total (n)**	**GIB events (no. of pts)**	**Females (%)**	**Mean age (yrs)**	**Cause of GIB**	**Assessment tool**	**Risk of bias**
Bombardier et al.	2000	USA, UK	8076	113	79.7	58	NSAIDs	Global Assessment of Disease Activity, Modified Health Assessment Questionnaire	Low risk of bias
Caradoc-Davies et al.	1984	New Zealand	531	39	.	> 75	NSAIDs	None	Moderate risk of bias
Chi et al.	2018	China	4728	928	40.1	72.5	NSAIDs	Mean Decrease Gini (MDG)	Low risk of bias
Myasoedova et al.	2012	USA	1626	458	68	55.9	Glucocorticoids, Upper GI disease, abdominal surgery	Malmo criteria	Low risk of bias
Silverstein et al.	1995	USA, Canada	5375	84	70.9	68	NSAIDs	Modified health Assessment Questionnaire	Low risk of bias
Matteson et al.	1995	USA	58	72 events	.	.	NSAIDs	.	Low risk of bias
Kim et al.	2015	Korea	117610	2184	55.6	73.8	NSAIDs	.	Low risk of bias
Tacheci et al.	2013	Czech Republic	37	3 (severe enteropathy)	81	55	NSAIDs	Capsule endoscopy, Mucosal damage Score (MDS)	Moderate risk of bias
Bombardier et al.	2000	USA, UK	8076	113	79.7	58	NSAIDs	Global Assessment of Disease Activity, Modified Health Assessment Questionnaire	Low risk of bias
Caradoc-Davies et al.	1984	New Zealand	531	39	.	> 75	NSAIDs	None	Moderate risk of bias
Chi et al.	2018	China	4728	928	40.1	72.5	NSAIDs	Mean Decrease Gini (MDG)	Low risk of bias
Myasoedova et al.	2012	USA	1626	458	68	55.9	Glucocorticoids, Upper GI disease, abdominal surgery	Malmo criteria	Low risk of bias
Silverstein et al.	1995	USA, Canada	5375	84	70.9	68	NSAIDs	Modified health Assessment Questionnaire	Low risk of bias
Matteson et al.	1995	USA	58	72 events	.	.	NSAIDs	.	Low risk of bias
Kim et al.	2015	Korea	117610	2184	55.6	73.8	NSAIDs	.	Low risk of bias
Tacheci et al.	2013	Czech Republic	37	3 (severe enteropathy)	81	55	NSAIDs	Capsule endoscopy, Mucosal damage Score (MDS)	Moderate risk of bias

### Publication Bias and Quality Assessment

Publication bias cannot be assessed since the number of included studies was less than 10. All studies had a low risk of bias, except for two studies which had a moderate risk of bias.^17, 22^

### Results of meta-analysis

Seven studies were selected to evaluate the prevalence of GI bleeding in adult RA patients.^16–23^ Bombardier et al. randomised RA patients into either the rofecoxib group or the naproxen group.^16^ Myasoedova et al. reported the incidence of upper and lower GI bleeding with the use of NSAIDs.^20^ Silverstein et al. randomised patients to receive either misoprostol or placebo and assessed GI bleeding with or without ulcer.^21^ A total of 20,431 patients were included in the analysis. There was a statistically significant risk of GI bleeding in RA patients receiving NSAIDs (prevalence = 2% (1%, 3%); P < 0.00001; I2 = 98%).

### Causes and outcomes related to gastrointestinal bleeding in rheumatoid arthritis patients

Bombardier et al. assessed the incidence of upper GI events in adult RA patients aged 50 or above receiving either non-selective or selective COX inhibitors. A total of 8076 patients were divided into two groups; one received rofecoxib (4047 patients) and the other received naproxen (4029 patients). The prevalence of GI bleeding was significantly lower (P < 0.001) in the rofecoxib group (31 events) as compared to those in the naproxen group (82 events). Moreover, the relative risk of GI events for patients in the naproxen group was significantly higher (RR 0.5; 95 % CI 0.3 to 0.6; P < 0.001) as compared to patients receiving rofecoxib. The mortality rate was 0.5% in the rofecoxib group and 0.4% in the naproxen group. A common cause of death was either a GI or cardiovascular event.^16^ Caradoc-Davies et al. included 531 elderly patients (75 years of age) on NSAID therapy to assess the incidence of upper GI bleeding.^17^ Among the 33 cases of GI bleeding, the common diagnosis was a duodenal ulcer, gastric ulcer, hiatal hernia, esophagitis, oesophageal varices, and Mallory-Weiss syndrome. Patients usually present with hematemesis, melena, pallor, or dyspnoea. Sixteen patients were diagnosed with RA and two cases of GI bleeding with concurrent use of NSAIDs were reported (16.6%). One patient with RA and not on NSAID therapy also suffered from GI bleeding. Subgroup analysis showed no association of gender, age, or steroid use with upper GI bleeding.^3^ Chi et al. conducted a retrospective study to see the risk factors associated with GI bleeding in patients prescribed NSAIDs ^18^. They included 4728 patients on NSAID therapy and found that 928 patients reported GI bleeding. Out of 4728 patients, 194 were diagnosed with RA. The interaction of RA patients on NSAID therapy with GI bleeding was nonsignificant (P = 0.349), as only 33 events were reported. However, rheumatic diseases other than rheumatoid arthritis like systematic lupus erythematosus, Sjogren syndrome, and gout were significantly associated with a higher risk of GI bleeding (P = 0.004). Multivariate analysis revealed the following independent risk factors linked with GI bleeding; family history of GI bleeding (OR 3.348; 95% CI 2.398 to 4.673; P = 0.000), peptic ulcers (OR 4.068; 95% CI 3.093 to 5.349; P = 0.000), history of cardiovascular and cerebrovascular disease (OR 1.476; 95% CI 1.181 to 1.845; P = 0.001), antiplatelet drugs (OR 3.106; 95% CI 2.574 to 3.748; P = 0.000), cholesterol level (OR 0.516; 95% CI 0.457 to 0.584; P = 0.000) and NSAIDs used for 0.5 to 3 months (OR 0.780; 95% CI 0.721 to 0.844; P = 0.000).^18^ Melena, hematemesis, antiplatelet drugs, cholesterol, and upper abdominal discomfort were the top-ranked factors according to the Mean Decrease Gini. Matteson et al. followed RA patients for up to 40 years, to see the trend in hospitalisation for GI bleeding. In this study, 58 patients were selected, and 46 patients reported symptoms of bleed. The incidence rate for GI bleeding was 0.52% per person-year. A total of 72 hospitalisations were seen and patients aged 53 or more were at a greater risk of hospitalisation. RA patients had a slightly higher rate of mortality as compared to other patients.^19^ Myasoedova et al. conducted a longitudinal population-based study to estimate the incidence of upper and lower GI events in RA vs non-RA patients.^20^

Before the cohort was incidence/index dated, RA was associated with a higher probability of any GI diagnosis (32%; p = 0.03), and upper GI event (21%; P = 0.04). During the follow-up of 10 years, RA group was associated with a significantly increased risk of developing any GI event (IR 3.64; 95% CI 3.10 to 4.25; RR 1.66; 95% CI 1.32 to 2.16), any upper GI event (IR 2.85; 95% CI 2.41 to 3.33; RR 1.72; 95% CI 1.35 to 2.20) and any lower GI event (IR 2.10; 95% CI 1.76 to 2.50; RR 1.47; 95% CI 1.13 to 1.91) as compared to the non-RA group. Upper GI bleeding was 1.71-fold (95% CI 1.12 to 2.67) more prevalent in the RA group as compared to the non-RA group (52 episodes in 813 patients). The incidence rate of upper GI bleeding in RA was IR 0.68 (95% CI 0.51 to 0.90). In this study, 229 RA patients died. Multivariate analysis revealed that upper GI bleeding and/or perforation are significantly associated with higher mortality (HR 2.1; 95% CI 1.5 to 3.0).^20^ Silverstein et al. investigated the effects of concurrent administration of misoprostol on serious upper GI complications in RA patients receiving NSAIDs.^21^ The number of patients receiving misoprostol was 4404 and the placebo group included 4439 patients. They found that complications were significantly reduced (OR 0.598; 95% CI 0.364 to 0.982; P = 0.049) among patients in the treatment group as compared to the placebo group. Patients reported a 51% reduction in serious ulcer complications in the misoprostol group (OR 0.487; 95% CI 0.268 to 0.886; P = 0.021). Gastric outlet obstruction was also significantly lower in misoprostol recipients (OR 0.101; 95% CI 0.013 to 0.787; P = 0.012). Logistic regression analysis revealed the following risk factors were significantly associated with an increased risk of GI complications; Age >75 years, peptic ulcer, history of GI bleeding, and cardiovascular disease. Mortality was similar (P > 0.20) between the two treatment groups and 14 patients died in the treatment cohort.^21^ Tacheci et al. evaluated the potential of endoscopy in diagnosing NSAID-induced enteropathy in RA patients.^22^ For this purpose, 37 patients were included in this study. Enteropathy was classified as mild (red spots or erosions), moderate (10–20 erosions), and severe (>20 erosion or ulcers or bleeding). Among the 25 patients diagnosed with enteropathy, 18 were classified as mild (49%), 4 as moderate (11%) and 3 as severe (8%).^23^ No correlation was established between enteropathy and RA activity (P = 0.710). Age and gender were insignificantly related to enteropathy. Kim et al. used a nationwide database to assess the risk of GI bleeding in diabetic patients aged ≥65 years.^12^ For this purpose, 117,610 patients were propensity-matched to exclude any confounding factor. 1384 episodes of GI bleeding were recorded in the 58,805 patients on NSAID treatment. The incidence rate ratio of GI bleeding was 1.68 (95% CI 1.54 to 1.84). The adjusted hazard ratio (aHR) of GI bleeding with NSAID use was 1.68 (95% CI 1.54 to 1.83). Liver disease (aHR 1.16; 95% CI 1.04 to 1.29), renal failure (aHR 1.16; 95% CI 1.04 to 1.29), and use of anti-coagulant medication (aHR 1.20; 95% CI 1.05 to 1.38) were significantly more common in patients experiencing GI bleed. From the NSAID group, 817 patients were diagnosed with RA. Despite the use of NSAIDs, the risk of GI bleeding was shown to be nonsignificant in RA patients, implying a potential differential risk profile compared to the general diabetic population. Furthermore, the study indicates a nonsignificant relationship between NSAID use and the incidence of cardiovascular events in RA patients, emphasising the complexity of the risk-benefit profile of NSAIDs in this population.^23^

**Figure 2.**
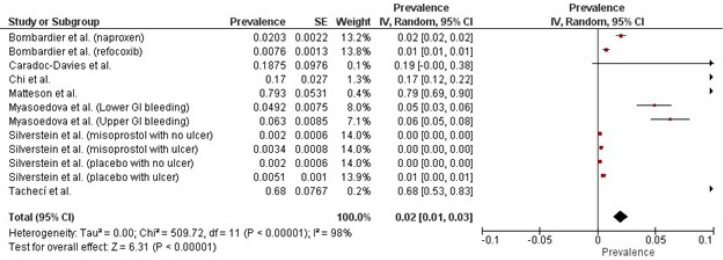
Prevalence of gastrointestinal bleeding in rheumatoid arthritis.

Overall, the common causes of GI bleeding discussed in the studies were age-related factors, cardiovascular events, history of GI complications, and peptic ulcers. The outcome varied by the use of specific NSAIDs and the presence of comorbidities.

**Table 2.** Quality assessment of the included cohorts.

**Study**	**Selection**				**Comparability**	**Outcome**			**Total Score**
	**Representation of exposed cohort**	**Selection of non-exposed cohort**	**Ascertainment of exposure**	**Outcome not present at the start of this study**		**Assessment of outcome**	**Length of follow-up**	**Adequacy of follow-up**	
Caradoc-Davies et al., 1984	1	1	1	1	0	1	1	1	7
Matteson et al., 1995	1	1	1	1	2	1	1	1	8
Myasoedova et al., 2012	1	1	1	1	2	1	1	1	8
Tacheci et al., 2013	1	1	1	1	0	1	1	1	7
Kim et al., 2015	1	1	1	1	2	1	1	1	8
Chi et al., 2018	1	1	1	1	2	1	1	1	8

## DISCUSSION

Acute GI bleeding is a medical emergency, often associated with significant morbidity and mortality and requires prompt management which includes immediate resuscitation, risk stratification, localisation of the source of bleeding, and appropriate intervention.^24^ While many cases may resolve spontaneously, some incidences of GI bleeding, particularly if acute, may cause significant hemodynamic instability which requires hospital admission and immediate resuscitative measures including aggressive fluid replacement and blood transfusions.^25^ Our findings shed light on the risk factors and outcomes related to GI bleeding in rheumatoid arthritis (RA) patients. Understanding the complex nature of GI problems in RA patients is critical for maximising clinical treatment methods and improving patient health. Guidelines for RA management often emphasise the necessity of comprehensive care, which includes monitoring and mitigating the potential side effects of disease-modifying antirheumatic medications (DMARDs) and nonsteroidal anti-inflammatory drugs (NSAIDs). While NSAIDs are often recommended to relieve pain and inflammation in RA patients, their usage is frequently associated with an increased risk of GI problems, as demonstrated in our study and supported by current literature.

Our systematic review and meta-analysis pooled data from 8 published studies shows that there is a significant risk of gastrointestinal bleeding in patients with rheumatoid arthritis. The risk can be attributed to multiple factors among which NSAID use and RA-associated vasculitis are the most prominent factors.

One of the most significant causes of GI bleeding in RA patients is RA-associated vasculitis i.e., inflammation of blood vessels which may involve small-, medium- or large-sized arteries. GI involvement due to rheumatoid vasculitis is a rare manifestation. Involvement of GI vessels by RA-associated vasculitis often results in debilitating GI symptoms including bleeding from the gut. Vasculitis can take the form of either occlusive or non-occlusive vessel disease. Occlusive injury can cause ischemic ulceration which may proceed to perforation. Non-occlusive disease can cause vascular leaking which can lead to oedema and haemorrhages.^26^ Vascular inflammation can cause localised as well as diffuse histopathological changes in the gastrointestinal tract which may result in mesenteric ischemia, ileus, perforation, stricture submucosal oedema, or haemorrhage depending on the extent of inflammation.^27^ Rheumatoid arthritis-associated vasculitis often presents with gastrointestinal symptoms such as abdominal pain, diarrhoea, nausea, vomiting, or gastrointestinal bleeding which might take the form of hematemesis, melena, or haematochezia.^28^

Many systemic autoimmune diseases, especially those involving soft tissues, such as systemic vasculitides, collagen vascular disease, Churg-Strauss syndrome, and Wegener’s granulomatosis take the form of gastrointestinal manifestations with bleeding from the GI tract a prominent feature.^29^ It is well-recognised that there is a close association between inflammatory arthritis and inflammatory bowel disease (IBD), and the occurrence of arthritis might anticipate the onset of bowel symptoms. As such, GI bleeding might be a manifestation of the associated IBD that has not yet manifested with other bowel symptoms.^30^

**Table 3.** Quality assessment of the included randomised controlled trials.

	**Sequence generation**	**Allocation concealment**	**Blinding of participants, etc.**	**Blinding of outcome assessment**	**Incomplete outcome data**	**Selective outcome reporting**	**Other sources of bias**	**Net risk**
Silverstein et al., 1995	Low	Unclear	Low	Low	Low	Low	Low	Low Risk
Bombardier et al., 2000	Low	Unclear	Low	Unclear	Low	Low	Low	Low Risk

Treatment of rheumatoid arthritis includes a multidisciplinary approach, with a major focus on controlling the ongoing inflammation by steroids and DMARDs and relieving pain with analgesics like NSAIDs. The advent of DMARDs has revolutionised the management of the condition by their remarkable role in controlling disease activity and preventing complications. However, the response and effectiveness are still limited in some patients, and this calls for the development of novel therapeutic targets and treatment modalities.^2^ Cases have been reported where concomitant use of DMARDSs and steroids led to pill-induced acute GI bleeding which responded to stoppage of the offending drug. The reason behind the bleeding was found to be pill-induced esophagitis, gastritis, and duodenitis on OGD.^31^ Moreover, it has been found that steroids increase the risk of GI bleeding more in hospitalised patients as compared to ambulatory patients.^32^ Additionally, it is crucial to differentiate between upper and lower GI bleeding, which have different causes and treatments. Upper GI bleeding is related to peptic ulcers and gastritis, is a major clinical issue since it can lead to serious consequences. While lower GI bleeding, is associated with diverticular disease and inflammatory bowel disease, and also require unique diagnostic and treatment strategies.^33^

NSAIDs have been traditionally used in the acute and chronic management of rheumatoid arthritis. It is well-recognised that NSAID use is associated with a significant risk of GI events and complications. The risk of peptic ulcer disease increases five times, and the risk of upper gastrointestinal bleeding increases four times with the use of non-selective NSAIDs. However, the incidence of GI toxicity is much lower with selective COX-2 inhibitors. Lower GI events are less well-characterised than upper GI events and may be associated with more critical consequences.^34^ Our study found that the risk of upper GI bleed is greater with NSAID monotherapy as compared to COX-2 inhibitor or low-dose aspirin monotherapy, and combination therapy generally increases the risk of bleeding. NSAIDs cause damage to the mucus lining of the gastrointestinal tract in the upper, middle as well and lower regions, and this results in bleeding that can either be concealed (presenting with unexplained iron deficiency) or conspicuous (presenting as melena, haematochezia or haematemesis). Symptoms such as dyspepsia and gastroesophageal reflux can be predictive of NSAID-induced peptic ulcer disease and its complications. Moreover, it has been found that co-administration of PPIs with NSAIDs can cause mucosal injury in the small bowel which may lead to occult bleeding.^35^

NSAID-induced enteropathy can lead to chronic occult gastrointestinal bleeding, and this can be accurately evaluated by non-invasive methods such as capsule endoscopy. However, there are no clinical and laboratory parameters that can precisely determine the presence or severity of NSAID-induced enteropathy.^22^ It has been seen that in addition to depending on the specific NSAIDs that are used, the risk of GI complications including bleeding also varies with the dosage of the drugs.^16^ The study by Chi et al. explores the complex relationship of risk factors associated with GI bleeding in patients prescribed NSAIDs. Notably, the study reports a lower likelihood of GI bleeding associated with NSAID use for 0.5 to 3 months (OR 0.780; 95% CI 0.721 to 0.844; P = 0.000), compared to other established risk factors such as a family history of GI bleeding or a history of peptic ulcers.^17^

NSAIDs cause injury to the gastroduodenal mucosal lining by different mechanisms which include direct irritating effects on the surface epithelium, dysfunction of the normal protective mucosal mechanisms such as impairment of prostaglandin synthesis, interruption of blood flow to mucosa, and loss of efficient injury repair mechanism.^36^ A study evaluating the risk of GI bleeding associated with concomitant use of NSAIDs, low-dose aspirin, and COX-2 inhibitors with other drugs found that various combinations of these drugs with corticosteroids, anticoagulants, SSRIs, and aldosterone antagonists generally increase the risk of gastrointestinal bleeding to variable extents. However, the greatest risk occurs with concomitant use of NSAIDs and corticosteroids.^37^

In a nutshell, patients with rheumatoid arthritis are at risk of upper as well as lower gastrointestinal bleeding events. This may be due to the associated vasculitis, but most often occurs due to the use of NSAIDs, DMARDs, and other drugs used in the management of the condition. Based on our study, we suggest that rheumatoid arthritis patients should be regularly followed up for evaluation of bleeding risk, and specifically the patients on NSAIDs, DMARDs, and biologics should be carefully monitored for adverse GI events including gastrointestinal bleeding.

## LIMITATIONS

Our study had some limitations: (a) only 2 RCTs were used; (b) evidence of multiple causes were not available in good quantity; (c) 2 studies had moderate risk of bias. Nonetheless, these articles were pivotal in conducting our manuscript.

## CONCLUSION

Our study showed that 2% RA patients were subjected to GI bleeding, when they used NSAIDS. Age-related factors, cardiovascular events, history of GI complications, and peptic ulcers were also related to GI bleeding. NSAID use and severity of bleeding resulted in variable comorbidities.
